# Induced Ripening Agents and Their Effect on Fruit Quality of Banana

**DOI:** 10.1155/2019/2520179

**Published:** 2019-05-02

**Authors:** S. D. T. Maduwanthi, R. A. U. J. Marapana

**Affiliations:** Department of Food Science and Technology, University of Sri Jayewardenepura, Nugegoda, Sri Lanka

## Abstract

Ripening is a genetically programmed highly coordinated irreversible phenomenon which includes many biochemical changes including tissue softening, pigment changes, aroma and flavour volatile production, reduction in astringency, and many others. Banana is one of mostly consumed fruit crops in the world. Since banana is a climactic fruit, induced ripening is essential in commercial scale banana cultivation and distribution to assure good flavour, texture, and uniform peel colour. Ethylene gas, acetylene gas liberated from calcium carbide, and ethephon are some of the commercial ripening agents used successfully in the trade and they have been widely studied for their effectiveness on initiating and accelerating the ripening process and their effect on fruit quality and health related issues. Lauryl alcohol was also shown as a ripening agent for bananas. Most studies suggest that there is no difference in biochemical composition and sensory quality in bananas treated with chemicals that induce ripening from naturally ripened bananas. However volatile profiles of artificially ripened bananas were shown to be considerably different from naturally ripened bananas in some studies. This review discusses induced ripening agents and their effect on fruit quality of bananas.

## 1. Ripening Physiology

The life of a fruit can be divided into three phases: fruit set, fruit development, and fruit ripening. Fruit ripening is the initiation of fruit senescence which is a genetically programmed highly coordinated process of organ transformation from unripe to ripe stage to yield an attractive edible fruit [1]. It is an irreversible phenomenon involving a series of biochemical, physiological, and organoleptic changes [2]. These changes include changes in carbohydrate content, increment of sugar content, changes in colour, texture, aroma volatiles, flavour compounds, phenolic compounds, and organic acids.

**Figure 1:**
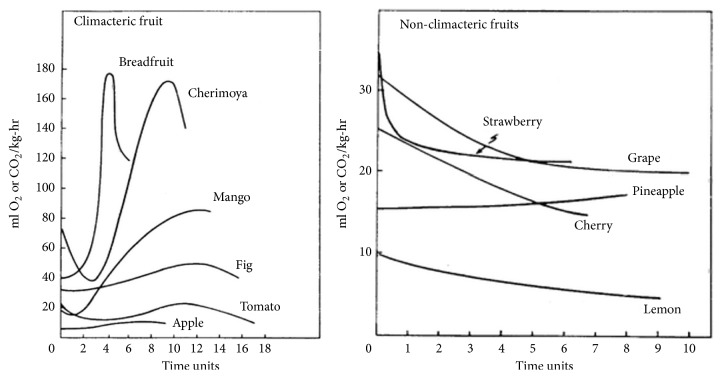
Respiratory pattern during ripening of climacteric and nonclimacteric fruits (source [10]).

Respiration is a process of breakdown of complex material in cells to simpler molecules giving energy and some specific molecules which are used in different cellular reactions. Thus respiration is a good indicator of cellular metabolic activity, and respiratory pattern is characteristic of the stages in the life cycle of a fruit such as development, ripening, and senescence [3]. Fruit ripening is closely linked to ethylene, a phytohormone that can trigger initiation of ripening and senescence. Based on regulatory mechanisms leading to fruit ripening, fruits can be divided into two groups: climacteric and nonclimacteric fruits.  Table 1 depicts classification of fruits as climacteric and non climacteric fruits. In climacteric fruits, as ripening proceeds there is a strong respiratory peak with high level of ethylene production. While in nonclimacteric fruits respiration rate is almost constant or shows a steady decline until senescence occurs, with little or no increase in ethylene production [4]. Therefore, climacteric fruits are referred to as ethylene dependent fruits and they have the capability to ripen after the harvest, often with the help of exogenous ethylene. However it is generally claimed that nonclimacteric fruits ripen only if they remain attached to the parent plant [5]. Respiratory pattern during ripening of several climacteric and non climacteric fruits are shown in the Figure 1.

The relationship between ethylene and fruit ripening has been studied for many decades. Although climacteric fruits are considered as ethylene dependent, it has been shown that in climacteric fruits some ripening changes occur independently of ethylene; also there are some changes in nonclimacteric fruits which are ethylene dependent. There are two systems of ethylene biosynthesis. System 1 represents ethylene biosynthesis during the preclimacteric stage of climacteric fruits as well as during the whole ripening process of nonclimacteric fruits. This is responsible for very low levels of ethylene production and is regulated in an autoinhibitory manner. System 2 operates during the ripening of climacteric fruit and is an autostimulated process which is responsible for high levels of ethylene production [6].

**Figure 2:**
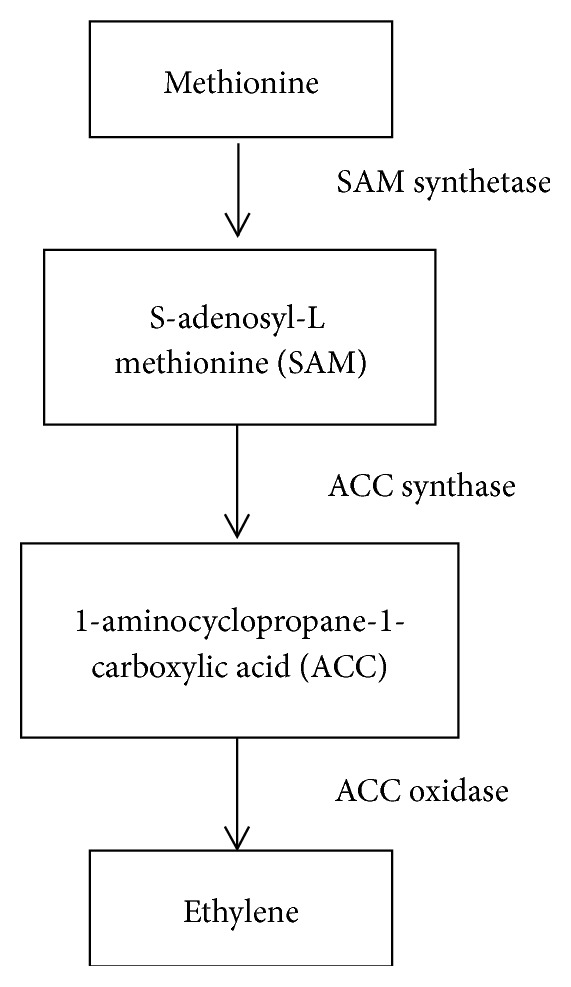
The mechanism of ethylene biosynthesis from methionine.

The ethylene biosynthesis pathway in higher plants has been well studied. Briefly, ethylene is synthesized from methionine. First methionine is converted to S-adenosyl-L methionine (SAM) which is catalyzed by SAM synthetase. Then SAM is converted to 1-aminocyclopropane-1-carboxylic acid (ACC). This conversion is catalyzed by the enzyme ACC synthase and then ACC is converted to ethylene catalyzed by ACC oxidase. Methionine is regenerated from ACC through the Yang cycle [1].  Figure 2 shows the mechanism of ethylene biosynthesis from methionine.

The Arabidopsis model system was used to study the mechanism involved in ethylene perception and signal transduction. However, more efforts in understanding the ethylene response during fruit ripening have focused on the characterization of tomato homologs. In summary, ethylene is perceived by receptors (ETR) in endoplasmic reticulum (ER). Ethylene receptors are multigene families encoding two types of closely related proteins, one subfamily with a histidine kinase domain and the other subfamily with a serine/threonine kinase function [7]. The receptors are negative regulators and in the absence of ethylene, constitutive Triple Response (CTR1) gets activated. CTR1 suppresses the ethylene response via inactivation of Ethylene Insensitive 2 (EIN2) [8]. This activates a transcriptional cascade, involving Ethylene Insensitive 3 (EIN3)/Ethylene Insensitive 3 like 1 (EIL1) as the primary transcription factor and then ERFs, which in turn regulate genes underlying ripening related behavior [8, 9].

## 2. Changes during Ripening

Compositional and structural changes occur during ripening leads to the fruit being desirable and edible. Among these changes, textural change is very important and a major event in fruit ripening. These textural changes differ with species where some fruits such as banana, mango, and papaya undergo substantial softening and fruits such as apple normally exhibit less softening. Textural changes and fruit softening are due to depolymerization and solubilization of cell wall components and loss of cell structure [12]. Change in turgor pressure and degradation of cell wall polysaccharides and enzymatic degradation of starch are determinant mechanisms of fruit softening. Cell wall polysaccharides such as pectin, cellulose, and hemicellulose undergo solubilization, deesterification, and depolymerization during ripening [13]. Cell wall degrading enzymes, such as pectin methylesterase, polygalacturonase, *β*-galactosidase, endo-1,4-*β*-d-glucanase, and many others, are involved in this mechanism. Further loss of neutral sugars and galacturonic acid followed by solubilization of the remaining sugar residues and oligosaccharides are also included in cell wall modifications [14]. According to gene expression analysis it has been revealed that ethylene directly regulates the transcription of both a softening-related PpPG gene that encodes an *α*-L-arabinofuranosidase/*β*-xylosidase (PpARF/XYL) and an expansin (PpExp3) [15]. In some fruits such as bananas which contain high level of starch in the fruit flesh, enzymatic hydrolysis of starch is a major factor in fruit softening. In citrus fruit, softening is mainly associated with change in turgor pressure, a process associated with the postharvest dehydration and/or loss of dry matter [11].

Colour development is an important maturity index of many fruits and associated with ripening. In many cases the colour change during fruit ripening is due to the unmasking of preexisting pigments by degradation of chlorophylls and synthesis of anthocyanins and carotenoids [16]. Carotenoid biosynthesis during ripening has been studied using tomato plant as a model. Carotenoids are derived from terpenoids and are synthesized in fruit at a high rate during the transition from chloroplast to chromoplast [4]. Anthocyanins are responsible for orange, red, pink, blue and purple colours in fruits and can be classified in to two groups as flavonoids and phenolic compounds [17]. They are synthesized in the cytosol and localised in vacuoles and synthesized via the phenylpropanoid pathway. Two classes of genes are required for anthocyanin biosynthesis, the structural genes encoding the enzymes that directly participate in the formation of anthocyanins and other flavonoids and the regulatory genes that control the transcription of structural genes [18]. It has been reported that ethylene is involved in regulation of genes related to anthocyanin biosynthesis [19].

Many fruits emit volatile compounds which are responsible for flavour and aroma of the certain fruit. The metabolism of fatty acids and branched amino acids act as precursors of aroma volatiles in fruit [20]. Aroma and flavour volatile profile of fruits mainly consist mainly of esters, alcohols, aldehydes, ketones, and terpenes. Many studies have been done to explain the association between volatile synthesis and ethylene production. Ethylene treatments can enhance the aroma volatile production in mangoes and honeydew melons [21, 22]. Further [23, 24] showed that aroma production is reduced when ethylene biosynthesis is inhibited by using aminoethoxyvinylglycine (AVG) or 1-methylcyclopropane (1-MCP) indicating that aroma synthesis is correlated with ethylene production and action in fruits.

Astringency which arises due to tannins in fruits shows a decreasing trend during ripening of many fruits. It is reported that astringency depends on the molecular structure of tannin which determines cross linking with proteins and glycoproteins [25]. Therefore tannins give astringent taste when they are dissolved in saliva. An increase in the molecular weight of tannin by polymerization which occurs during ripening causes a lack of astringency due to the insolubility of tannins [26].

## 3. Introduction to Banana

Banana (*Musa *spp.) is one of the most widely cultivated and widely consumed fruit crops in the world. It is said to be as one of the earliest fruit crops which is cultivated at the beginning of the civilization. Bananas are native to South East Asia and it is cultivated in over 130 countries throughout the tropical and subtropical regions of the world [27]. It is recorded as the fourth largest food crop of the world after rice, wheat, and maize [28]. The annual world production of bananas is around 114 million metric tons from an area of 5.6 million ha (FAO 2018). From this total banana production about 50% is consumed in cooked form which is often term plantains while the rest is dessert types [29]. Brazil, India, and Philippines are the principal countries in terms of production of bananas.

Bananas grow in a large range of environments and can produce food all year round. In Sri Lanka, nearly 60,000 ha (20,000ha and 40,000ha in wet zone and dry and intermediate zones respectively) of land is under banana cultivation. It covers about 54% of the total fruit lands. 13,000ha is used to cultivate plantain (for curries) and other 47,000ha is used to cultivate dessert type [30]. There were twenty-nine banana cultivars and two wild species in Sri Lanka [31]. According to a case study in Sri Lankan crop sector [32] currently there are 55 local cultivars in Sri Lanka. Some plantain types of bananas identified in Sri Lanka include* Alukesel* (ABB),* Mondan* (ABB),* ElaMondan, Atamuru, and Kithala*. The most popular dessert type bananas in Sri Lanka are* Embul* or* Ambul* (Mysore AAB),* kolikuttu *(Silk AAB),* Anamalu* (Gros Michel AAA),* Binkehel* (Dwarf AAA),* Rathambala* (AAA), and* Ambon* (AAA) [33]. According to the Department of Agriculture, many banana varieties grow freely all over Sri Lanka, all year round. They are cultivated in large, medium, and small-scale orchards, and in home gardens. According to Agricultural statistics in Sri Lanka, 2015, the total production of banana is nearly 530, 124 MT annually.

## 4. Induced Ripening of Bananas

Fruit ripening is a combination of physiological, biochemical, and molecular processes leading to changes in pigments, sugar content, acid content, flavour, aroma, texture, etc. Since banana is a climacteric fruit it is usually harvested at the preclimacteric stage and for commercial purposes artificially ripened. Artificial ripening enables traders to minimize losses during transportations as well to timely release the product at desired ripening stage. Bananas can be artificially ripened using different ripening agents.

### 4.1. Ethylene Gas

The most popular method practiced in developed countries is application of ethylene gas in ripening rooms. Modern banana ripening rooms are designed with techniques to control temperature, humidity, and ethylene gas concentration and they are equipped with proper ventilation and exhausting systems. Banana combs are properly packed and kept in these rooms and then ethylene is supplied at proper temperature and humidity. Mostly “catalytic generators” are used to generate ethylene in commercial ripening rooms. The concentration of ethylene required for different commodities to enhance ripening is different.

However early in the banana history many researchers tested ethylene as an induced ripening agent. Von Loesecke [34] commented that some scientists showed that ripening of bananas can be induced by exposing them to vapour of apples, which was confirmed when it was shown that ripening of bananas can be accelerated using the vapour that had previously passed through ripe bananas. Finally, it was concluded that exogenous ethylene treatment can induce ripening of bananas with increased rate of respiration and increased level of endogenous ethylene [35, 36]. Burg and Burg [37] reported that low ethylene concentration as 0.1ppm is effective in accelerating the ripening of bananas. Dominguez and Vendrell [36] showed that exogenous ethylene, 100ppm treatment for 12 hours, can immediately increase the endogenous ethylene and CO_2_ production similar to respiratory climacteric. Further this study showed that increment in respiration depends on time of treatment whereas 12-hour treatment was slightly effective than 6 hours treatment. Lohani [38] noted 11-fold decrease in fruit firmness which occurred within two days after exogenous ethylene treatment. Further it was recorded that ethylene treatment regulated up the activity of four cell wall hydrolases, pectin methyl esterase, polygalacturonase, pectate lyase, and cellulose in “Dwarf Cavendish” bananas.

### 4.2. Acetylene

Calcium carbide when hydrolysed produces acetylene which is an ethylene analogue. Mostly in developing countries including Sri Lanka, calcium carbide is widely used for artificial ripening of bananas, though it is prohibited by the government regulations. Hartshorn [39] conducted a series of experiments to identify effects of acetylene on ripening process of bananas. It was shown in this study that acetylene emits from calcium carbide can enhance banana ripening as treated fruits were uniformly yellow with good flavour, medium starch content, and comparatively soft texture after 120 hours while control samples were remain unripe after same period of time. According to Burg and Burg [37] acetylene has a lower biological activity than ethylene and it was reported in this study that concentration of acetylene should be 2.8 ml/L to enhance ripening of bananas. However Thompson and Seymour [40] reported that bananas do not respond to acetylene at 0.01ml/L while the treatment with acetylene in 1 ml/L led to indistinguishable colour and soluble solids content compared to those ripened by exposure to ethylene at same concentration. Further it was shown in this study that there is no significant difference in sensory attributes between bananas treated with ethylene and acetylene at 1ml/L when they are compared at same stage of ripeness.

However, calcium carbide is not generally recognized as safe [41] and prohibited in Sri Lanka as in most countries under Section 26 of the food (labelling and miscellaneous) regulation of 1993. Calcium carbide is considered as hazardous due to several reasons. Commercial calcium carbide contains traces of arsenic and phosphorous hydride [42, 43] and acetylene emitted from commercial calcium may also contain up to 3 ppm arsenic and up to 95 ppm phosphorous hydride [42, 44]. Arsenic and phosphorous hydride can be poisonous to humans and cause vomiting, diarrhoea with or without blood, burning sensation of the chest and abdomen, thirst, weakness, difficulty in swallowing, irritation or burning in the eyes and skin, permanent aye damage, and so on [42]. Exposure to acetylene gas can cause headache, vertigo, dizziness, delirium, seizure, and even coma [43].

### 4.3. Ethephon

Ethephon (2-chloroethylphosphonic acid), an ethylene releasing compound, is categorized as noncarcinogenic to humans by IARC (International Agency for Research on Cancer). It penetrates into the fruit and decomposes to ethylene [45] and has been shown to hasten ripening of several fruits including bananas, apples, tomatoes, mango, peaches, citrus fruits, and guava [46–48]. Pendharkar [49] treated bananas with different concentrations of ethephon. Here it was found out that different concentrations of ethephon significantly influence chemical changes during ripening and 1000 ppm was found as the best concentration of ethephon for early ripening. Nair and Singh [50] showed that prestorage treatments of mangoes (*Mangifera indica *L., cv. Kensington Pride) with ethephon (500 mg/L) for five minutes increased TSS, TSS/acid ratio, and sugars and reduces chilling injury. Adane [51] compared ethephon treatment and traditional kerosene smoke treatment and their effect on ripening of “Cavendish” bananas, where it was shown that ethephon treated fruits demonstrated higher sensory quality. Apart from being used to initiate ripening, ethephon has been recorded as plant growth regulator which can be used to increase fruit size, induce flowering, enhance colour, and induce flower abscission [48].

It has been reported that health related studies on ethephon has shown that it has hepatotoxic potential. Ethephon is an organophosphorus compound and it has been reported to get rapidly absorbed in the gut. There is a possibility of converting ethephon into ethylene oxide, then to ethanediol and hydroxyethyl-glutathione and mercapturic acid. Further it has been studied that it can inhibit the growth Streptomyces and their antibiotic production [52]

### 4.4. Other Ripening Agents

Ethylene glycol is C_2_H_6_O_2_ and commonly used as a coolant and antifreeze. Goonatilake [53] experiments on the effectiveness of ethylene glycol as a fruit ripening agent have reported that when diluted with water, various fruits will ripen faster in colder climactic conditions. According to Stahler and Pont [54] bananas can be artificially ripened by using alkyl alcohol containing between 6 and 14 carbon atoms. Further, it has been reported, in this patent, that lauryl alcohol is preferred to ripen green bananas and treatment with 0.01% by the weight of bananas can change bananas to completely yellow within 48 hours without any loss of palatability.

One of the traditional methods used in Sri Lanka, as well as in many other countries, is smoking. In Sri Lankan traditional practice, bananas are laid in a pit, covered with banana leaves or a sheet cover and smoke, generated from burning of semidried leaves, is directed into the pit. In some countries kerosene burners are used to generate smoke in commercial scale banana ripening. Smoke is known to accelerate ripening due to the presence of acetylene, ethylene, and other unsaturated compounds which can enhance ripening [55, 56].

Some other ripening agents are ethanol, methanol, propylene, and methyl jasmonate [57, 58]. Ethylene is also emitted from fruit that have already initiated to ripen and can be used to enhance ripening of other fruits. Ethylene production can be stimulated by mechanically wounding the fruit. Here ethylene production is directly proportional to wound dimensions [59].

## 5. Effect of Induced Ripening Agents on Quality of Bananas

Lustre [60] compared physicochemical changes occurring during natural ripening and acetylene induced ripening in “Saba” bananas. Here it was shown that acetylene affects the rate of chemical changes during ripening; nevertheless it did not significantly affect the final levels of sugar and starch content in the ripe pulp. Another study which was conducted to examine comparative effect of acetylene and ethylene gas on banana ripening showed that fruits treated with acetylene at 1ml/L exhibited the similar colour score and soluble solids content in fruits treated with ethylene gas. However in this study it was further recorded that sensory quality is the same in the fruits treated with ethylene gas and acetylene gas when compared at same stage of ripeness [40]. Similar research was conducted by Sarananda [55] where effect of acetylene liberated from calcium carbide on ripening of “*Embul*” (*Musa acuminata*, AAB) banana. It was clearly exhibited in this study that naturally ripened fruits have excellent sensory quality with flesh colour, flavour, taste, and overall acceptability compared to calcium carbide treated fruits at the stage of fully yellow (Colour Index-6). Nevertheless it was shown that calcium carbide treated fruits achieved the same sensory quality when they were kept for one day more after reaching the fully yellow stage.

According to Adane [51] smoking bananas caused deep yellow colour with black spots on the peel with over softening while ethephon treatment gave uniform yellow colour in the peel at the end of the ripening period. As well Islam* et al*., 2018, showed that bananas treated with kerosene smoke have significantly low levels of vitamin C. Further in this study it was revealed that calcium carbide treated bananas shows high level of sulphur and trace amount of arsenic and phosphorous.

Gandhi [61] compared natural ripening agents including apple, pear, and tomato with calcium carbide. In this study it was revealed that apple is an effective ripening agent compared to calcium carbide and other tested natural agents and it contributed to the highest sensory acceptability. However calcium carbide treated fruits exhibited the least organoleptic quality compared to naturally ripened and ethephon treated bananas in [62]. Further in this study, the best physicochemical quality attributes such as total sugar, vitamin C, titratable acidity, pH, and total soluble solids were recorded in naturally ripened banana compared to artificially ripened bananas, whereas controversial results were obtained by Kulkarni* et al*., 2011, which showed that sensory quality and other physicochemical quality attributes were excellent in ethephon (500-1000 ppm) treated bananas compared to naturally ripened fruits at the 6^th^ day of storage. Reference [63] compared ripening techniques such as ethephon, smoking, and keeping in low density polyethylene plastic, in* Teff* straw, and in banana leaves. The results supported that smoking enhance faster ripening but led to least marketability, 28.67% at 10^th^ day of storage when other treatments reported more than 83% of marketability. The reduction of marketability of smoked bananas was due to blackening and over softening.

Effect of induced ripening agents including calcium carbide, potash, and leaves of* Irvingia gabonensis* and* Jatropha curcas *leaves on nutritional and mineral composition of bananas was studied by Sogo-Temi* et al*. [64]. They reported that the chemical ripening agents tested contributed to lower levels of protein compared to biological ripening agents. Also levels of Pb, Cu, Zn, and Mn were higher in calcium carbide treated fruits than other ripening agents.

Hakim [65] found that nutritional value of ethephon treated bananas is less than untreated samples where ascorbic acid content, *β*-carotene content, and mineral content were less in quantities.

Reference [66] compared aroma compounds in naturally ripened and ethylene treated ripened banana (*Musa acuminata*). According to the data obtained by this study total aromatic concentration was higher in naturally ripened bananas (60 437 *μ*g/kg) compared to that (55,243 *μ*g/kg) of ethylene treated bananas. Most of ester compounds including n-butyl acetate, isopentyl isobutanoate, 2-pentyl formate, 2-cyclohexanol acetate, ethyl isobutanoate, and 2-pentenyl butanoate were detected in significantly higher levels in naturally ripened bananas compared to ethylene treated samples. The same study showed that there was a significant difference in sucrose level as it was 98.40 g/kg in naturally ripened banana and 89.40 g/kg in ethylene treated bananas. As well total polyphenols content were reported as significantly different in the two types where naturally ripened bananas had 24.90 GAE, mg/L while ethylene treated bananas had 25.10 GAE, mg/L level of total polyphenols.

## 6. Conclusions

The banana ripening process can be enhanced using artificial ripening agents such as ethylene gas, ethephon, acetylene (emitted from calcium carbide), ethylene glycol, and alkyl alcohols (containing 6-14 carbon atoms such as lauryl alcohol). Smoke generated from burning green leaves or kerosene burners are also used as traditional methods in banana ripening. Many studies on the effect of different ripening agents on fruit quality appear to show that naturally ripened bananas exhibit better sensory characteristics compared to treated fruits.

## Figures and Tables

**Table 1:** Classification of fruits as climacteric and nonclimacteric fruits (source [4, 11]).

Climacteric fruits	Nonclimacteric fruits
Apple	Cashew

Apricot	Cherry

Avocado	Cucumber

Banana	Grape

Blueberry	Grapefruit

Durian	Lemon

Guava	Lime

Kiwifruit	Litchi

Mango	Mandarin

Papaya	Melon

Passion fruit	Orange

Peach	Pineapple

Pear	Pomegranate

Persimmon	Rambutan

Plum	Raspberry

Sapodilla	Strawberry

Tomato	Watermelon

## Data Availability

The numerical data supporting this review article are from previously reported studies and data sets, which have been cited, and are available from the corresponding author upon request.
